# The importance of MHC class II in allogeneic bone marrow transplantation and chimerism-based solid organ tolerance in a rat model

**DOI:** 10.1371/journal.pone.0233497

**Published:** 2020-05-22

**Authors:** Kai Timrott, Oliver Beetz, Felix Oldhafer, Jürgen Klempnauer, Florian W. R. Vondran, Mark D. Jäger

**Affiliations:** Department of General, Visceral and Transplant Surgery, Hannover Medical School, Hannover, Germany; Centro Cardiologico Monzino, ITALY

## Abstract

Mixed hematopoietic chimerism enables donor-specific tolerance for solid organ grafts. This study evaluated the influence of different serological major histocompatibility complex disparities on chimerism development, graft-versus-host disease incidence and subsequently on solid organ tolerance in a rat model. For bone marrow transplantation conditioning total body irradiation was titrated using 10, 8 or 6 Gray. Bone marrow transplantation was performed across following major histocompatibility complex mismatched barriers: complete disparity, MHC class II, MHC class I or non-MHC mismatch. Recipients were clinically monitored for graft-versus-host disease and analyzed for chimerism using flow cytometry. After a reconstitution of 100 days, composition of peripheral leukocytes was determined. Mixed chimeras were challenged with heart grafts from allogeneic donor strains to define the impact of donor MHC class disparities on solid organ tolerance on the basis of stable chimerism. After myeloablation with 10 Gray of total body irradiation, chimerism after bone marrow transplantation was induced independent of MHC disparity. MHC class II disparity increased the incidence of graft-versus-host disease and reduced induction of stable chimerism upon myelosuppressive total body irradiation with 8 and 6 Gray, respectively. Stable mixed chimeras showed tolerance towards heart grafts from donors with MHC matched to either bone marrow donors or recipients. Isolated matching of MHC class II with bone marrow donors likewise led to stable tolerance as opposed to matching of MHC class I. In summary, MHC class II disparity was critically associated with the onset of graft-versus host disease and was identified as obstacle for successful development of chimerism after bone marrow transplantation and subsequent donor-specific solid organ tolerance.

## Introduction

Stable mixed chimerism after allogeneic bone marrow transplantation (BMT), defined as coexistence of donor and recipient hematopoietic cells, is associated with donor-specific tolerance towards solid organ grafts [1]. This has been demonstrated in divergent animal models and selected patients in the past [2–5].

Limitations for the clinical use of mixed chimerism in solid organ transplantation are side effects like toxicity due to conditioning of the recipient, risk of engraftment failure and graft-versus-host disease (GvHD) [6]. It is therefore crucial to further investigate mechanisms of optimal cytoreductive conditioning and induction of stable mixed chimerism in experimental models.

Since cytoreductive conditioning of the bone marrow recipient, by total body irradiation (TBI) or immunosuppressive regimens, is necessary to create an environment for competing donor-derived hematopoietic stem cells, we have investigated the efficacy of different conditioning strategies in experimental rat models in the past [7–9].

In this study, we investigated the effect of different TBI dosages on the incidence and severity of GvHD and on hematopoiesis to discriminate between myeloablative and non-myeloablative (myelosuppressive) conditioning regimens in a congeneic rat model.

We have demonstrated the importance of major histocompatibility complex (MHC) class II antigens in solid organ transplantation in general as well as in donor-specific tolerance towards solid organ grafts in chimeric recipients with a near to total T cell depletive conditioning regime in our previous work [10,11]. We therefore aimed to further elucidate the effect of serological MHC (designated as RT1 system in the rat) disparities on induction of chimerism and incidence of GvHD in bone marrow recipients, conditioned by a varying degree of TBI [12,13].

Furthermore, we performed allogeneic heart transplantation in high-grade chimeras after a reconstitution period of 100 days to evaluate donor-specific tolerance depending on different MHC disparities.

Our results demonstrate the significance of MHC class II for efficient and safe induction of stable chimerism and consecutive organ tolerance in a congenic rat model.

## Material and methods

### Ethics

All animal procedures were approved by the Ethics Animal Review Board of the regional authorities for consumer protection and food safety of Lower Saxony (LAVES, Oldenburg, Germany) (Approval ID 02/528).

### Animals

Male inbred rats weighing 200 to 300 grams (aged 6 to 8 weeks) were purchased from and maintained in the central animal facility of Hannover Medical School, Hannover, Germany. An overview of the used strains on Lewis (LEW) background and the corresponding MHC immunogenetics is given in **Table 1**. Brown Norway rats (RT1^n^) were used as 3^rd^ party heart donors (see below) and were purchased from Charles River Laboratories (Sulzfeld, Germany).

**Table 1:** Rat strain combinations for BMT and corresponding MHC (RT1 regions) disparities.

MHC disparity	Donor	RT1A class I	RT1B/D class II	RT1C/E class I	Recipient	RT1A class I	RT1B/D class II	RT1C/E class I
Complete	LEW.1W	u	u	u	LEW.1A	a	a	a
MHC II	LEW.1AR1	a	u	u	LEW.1AR2	a	a	u
MHC I	LEW.1AR1	a	u	u	LEW.1WR1	u	u	a
non-MHC	LEW.1W	u	u	u	LEW.1U-7B	u	u	u

BMT, bone marrow transplantation; MHC, Major histocompatibility complex.

Animal housing was provided in a specific pathogen free facility under a circadian rhythm of light and dark cycle with free access to water and food.

For anesthetics we applied ketamine (intraperitoneal; 100 mg/kg body weight) and lidocaine (0.5%; intraincisional; 5 mg/kg per body weight). Metamizole (1 g) was added to 500 ml of drinking water immediately after the operation.

In the first four weeks after heart transplantation (see below), recipients were visited and heart grafts were palpated daily, in the further postoperative course graft palpation was performed every two days to evaluate graft function. After graft rejection or at the end of follow-up of 100 days, recipients were sacrificed by carbon dioxide inhalation and subsequent cervical dislocation. Of note, this euthanizing procedure was used for all animals that acquired a poor clinical state post TBI and BMT or post heart transplantation (amongst others defined by loss of activity, increased sleepiness and reduced food intake).

### Total body irradiation

Healthy rats were gamma-irradiated with a single dose of either 10, 8 or 6 Gray (Gy) TBI with a linear accelerator (Philips MU15F/225 kV, Hamburg, Germany) one day before BMT (defined as day -1).

### Bone marrow transplantation

Bone marrow cells were harvested from long-bones of euthanized donor rats. Mature α/β TCR^+^ cells were removed *in vitro* from bone marrow cell inocula using mouse anti-α/β TCR monoclonal antibody (mAb) (R73, mouse IgG_1_, provided by K. Wonigeit, Hannover) and immunomagnetic beads (Dynabeads M-450, goat anti-mouse IgG, Dynal, Great Neck, NY). 1 x 10^8^ nucleated bone marrow cells were intravenously injected per recipient one day after TBI (defined as day 0).

### Assessment of GvHD

All animals were evaluated for clinical manifestation of GvHD every five days for two months after BMT via severity scoring using GvHD specific criteria for rodents [14]. The criteria comprised weight loss (> 10% to < 25%: grade 1, > 25%: grade 2), posture (hunching noted at rest: grade 1, severe hunching impairing movement: grade 2), activity (mild to moderately decreased: grade 1, stationary unless stimulated: grade 2), fur texture (mild to moderate ruffling: grade 1, severe ruffling / poor grooming: grade 2) and skin integrity (scaling of paws / tail: grade 1, obvious areas of denuded skin: grade 2). At the time of analysis each animal was graded for each criterion and an animal specific score was calculated. A clinical GvHD index per group was subsequently generated as mean score per day of all single animal scores.

### Heart transplantation

Heart transplantation was performed on day 100 after BMT. Heart grafts were explanted, immediately perfused *ex vivo* with heparinized isotonic saline solution and stored in ice cold water. Subsequently, heart grafts were heterotopically (intraabdominally) transplanted by performing anastomoses between donor ascending aorta and the recipient abdominal aorta as well as the donor pulmonary trunk and the recipient inferior vena cava via 8–0 monofilament suture, as previously reported [15–17]. The total (cold and warm) ischemia time of the graft did not exceed 60 minutes. Completed graft rejection was defined as complete cessation of palpable heartbeat. Of note, donor type and 3^rd^ party grafts were not implanted into the same recipients, but were instead allocated to the respective groups (see also **S1 Table**).

### Cell preparation

Lymph nodes and spleens were gently minced on a 70 μm stainless steel sieve. The resulting cell suspensions as well as heparinized blood were rendered free of red blood cells by an ammonium chloride solution (155 mM NH 4 Cl, 10 mM K HCO_3,_ 0.1 mM sodium EDTA). Blood cell counts were conducted by hematology analyzer (Sysmex XE2100, Diamond Diagnostics, USA). Lymph node cells and spleen cell numbers were counted in a Neubauer counting chamber.

### Flow cytometry

For primary staining of blood leukocytes, natural killer cells (NK cells) and granulocytes the following monoclonal antibodies were used: anti-α/β TCR mAb (R73, mouse IgG_1_), PE-conjugated anti-CD4 mAb (W3/25, mouse IgG_1_; both generously provided by K. Wonigeit, Hannover), PerCP-conjugated anti-CD8a mAb (OX-8, mouse IgG_1_, BD Pharmingen), anti-CD45 mAb (OX1, mouse IgG_1_, BD Pharmingen), anti-CD45RA mAb (OX-33, mouse IgG_1_, BD Pharmingen), anti CD103 mAb as marker for dendritic cells in rats (OX-62, mouse IgG_1_, BD Pharmingen), FITC-conjugated anti-MHC class II mAb (OX-6, mouse IgG_1_, BD Pharmingen) or biotin-conjugated anti-RT1B/D^u^ mAb (1H1A, rat IgG_1;_ provided by K. Wonigeit, Hannover), FITC-conjugated anti-RT7.2 mAb (HIS 41, mouse IgG_1_, BD Pharmingen), biotin-conjugated anti-NKR-P1 (3.2.3, mouse IgG_1;_ provided by K. Wonigeit, Hannover) and biotin-conjugated HIS48 (mouse IgG_1;_ provided by K. Wonigeit, Hannover) for granulocytes staining.

For detection of donor chimerism of full MHC disparity and MHC II disparity the “u” haplotype of donor-derived fractions (1H1A) from MHC class II expressing cells (OX-6) within CD45^+^ leukocytes (OX-1) was used. For detection of donor chimerism of non-MHC disparity the “a” haplotype of donor-derived fractions (RT7.2) from MHC class II expressing cells (OX-6) within CD45^+^ leukocytes (OX-1) was used. For detection of donor chimerism of MHC I disparity the “a” haplotype of donor-derived fractions (RT1.Aa) from MHC class I expressing cells (OX-6) within CD45^+^ leukocytes (OX-1) was used.

Secondary stainings were performed with FITC-conjugated or PE-conjugated goat anti-mouse antibodies (BD Pharmingen). Blocking was performed with normal mouse serum. As fluorochromes streptavidin-PE or streptavidin-APC (BD Pharmingen) were used. Staining with Propidium iodide was implemented to exclude dead cells (PI, Sigma Chemical Co., St. Louis, MO). All staining steps were performed on ice (4° Celsius). Flow cytometry was performed using a FACS Calibur (Becton Dickinson, Mountain View, CA).

### Histology

For analysis of lymphocyte infiltration frozen sections of heart grafts embedded in Tissue-Tek (Sakura, Alphen aan den Rijn, Netherlands) were air dried, acetone/methanol fixed, and incubated with following monoclonal antibodies: anti-α/β TCR (R73), anti-CD4 (W3/25), anti-CD8 (OX-8) and anti-CD25 (OX-39, Bio-Rad AbD Serotec, Puchheim, Germany). Antibodies were purified in our laboratory except where noted. Stained cells were detected with bridge antibodies (rabbit anti-mouse Ig) and alkaline phosphatase anti-alkaline phosphatase (APaAP) (both Dako, Hamburg, Germany). Nuclear staining was performed with hematoxylin (Merck, Darmstadt, Germany). Cells were counted per high-power field (200-fold magnification).

### Statistics

Results in the text, figures and tables are presented as mean ± standard deviation, depicting at least five animals per group, unless stated otherwise.

Statistical analyses were performed using IBM SPSS Statistics 26.0 (Somers, NY USA) applying the unpaired and paired *t* test for comparison of mean values and the log-rank test for comparison of graft survival. Graphics were created via GraphPad Prism 8.0 (La Jolla, CA USA). Significance is indicated with * for p-values ≤ 0.05, ** for p-values ≤ 0.005 and *** for p-values ≤ 0.001. If no asterisk is shown in the respective graph or table, the results were not significantly different.

## Results

### Effect of serological MHC disparities in BMT conditioned by different total body irradiation dosages

In this study, conditioning prior to BMT was performed by different dosages of TBI without any further chemotherapy or immunosuppression. Clinical screening revealed 10 Gy as myeloablative dosage causing death by aplasia between days 12 to 15 (n = 6). Although 8 Gy TBI profoundly suppressed hematopoiesis indicated by anemia and mucosal bleeding between weeks 1 to 3, myeloablation within the following 100 days was not observed. 6 Gy TBI did not suppress hematopoiesis nor cause any deaths during follow up.

To assess the influence of different serological MHC disparities on chimerism development, BMT was performed across following MHC barriers: complete MHC disparity, solely MHC class II, solely MHC class I or non-MHC disparity (see also **Table 1**).

Recipients were irradiated with 10, 8 or 6 Gy on day -1 and were screened for donor-derived cells in peripheral blood on days 30 and 100 post BMT by flow cytometry. Chimerism was regarded as stable when definitively detectable on day 100.

After myeloablative conditioning with 10 Gy all surviving animals showed high-grade chimerism independent from the grade of MHC disparity (**Table 2**).

**Table 2:** Incidence and degree of chimerism after BMT according to TBI dosage and MHC disparity.

TBI	MHC	BMT	Chimerism				Death
dosage (Gy)	disparity	n	incidence		%		n
			day 30	day 100	day 30	day 100	
10	Complete	16	15 / 15	12 / 12	91 +/- 4	90 +/- 2	4
	Class II	20	18 / 18	15 / 15	87 +/- 7	81 +/- 4	5
	Class I	18	18 / 18	18 / 18	93 +/- 3	95 +/- 2	-
	None	10	10 / 10	10 / 10	94 +/- 4	93 +/- 2	-
8	Complete	18	6 / 18	3 / 15	39 +/- 28	10 +/- 6	3
	Class II	9	4 / 9	2 / 7	42 +/- 27	12 / 23	2
	Class I	10	9 / 10	8 / 10	58 +/- 19	65 +/- 27	-
	None	6	6 / 6	6 / 6	84 +/- 8	87 +/- 5	-
6	Complete	6	0 / 6	0 / 6	-	-	-
	Class II	6	0 / 6	0 / 6	-	-	-
	Class I	6	2 / 6	1 / 6	16 +/- 3	13	-
	None	8	8 / 8	8 / 8	63 +/- 5	68 +/- 7	-
No TBI	None	6	0 / 0	0 / 0	-	-	

BMT, bone marrow transplantation; MHC, Major histocompatibility complex.

After myelosuppressive conditioning by 8 Gy of TBI the engraftment rate of donor hematopoietic stem cells on day 100 was lower in case of complete MHC disparity (3 of 15 animals, mean chimerism degree of 10 +/- 6%) or isolated MHC class II disparity (2 of 7 animals, chimerism degrees of 12 and 23%) when compared to engraftment upon isolated MHC class I mismatch (8 of 10 animals, mean chimerism degree 65 +/- 27%). Recipients receiving BMT from donors with isolated non-MHC disparity did not show any restriction in hematopoietic stem cell engraftment rate (6 of 6 animals, mean chimerism degree 87 +/- 5%). Upon 6 Gy of TBI stable chimerism in case of complete MHC or isolated MHC class II disparity of BMT donor was not found in any of the 6 recipients, respectively, whereas only 1 of 6 recipients receiving BMT with isolated MHC class I disparity showed scarce engraftment (13%) on day 100. An unrestricted engraftment (8 of 8 animals) with slightly reduced chimerism degrees (68 +/- 7%) was observed in case of BMT donors with isolated non-MHC disparity.

### BMT side effects depending on MHC disparity after high dosage total body irradiation conditioning

**Fig 1:**
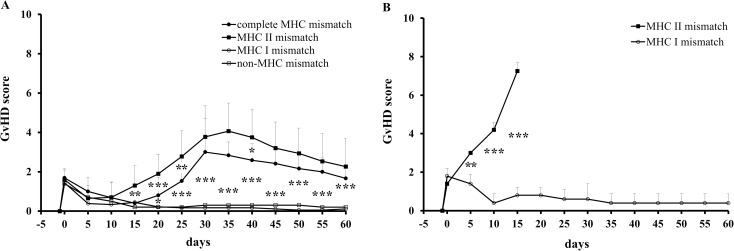
GvHD scores after TBI (10 Gy) and BMT across different MHC disparities. (A) After BMT (with T-cell depletion) complete MHC or isolated MHC class II mismatch led to significantly higher GvHD scores over the course of time. (B) After BMT without T-cell depletion and additional donor lymph node cell injection a further increase of GvHD scores was observed in case of isolated mismatch of MHC class II as opposed to mismatch of MHC class I.

Survival rates after BMT with 10 or 8 Gy of TBI were inferior in case of complete MHC or isolated MHC class II disparity: Death by aplasia after myeloablative conditioning was only observed upon complete MHC disparity in one of the recipients on day 14 and after engraftment failure of BM with isolated MHC class II disparity in two recipients on days 22 and 24, respectively. Death due to extensive GvHD symptoms following complete MHC disparity occurred in 3 of 16 animals after 10 Gy (days 30, 31 and 34) and in 3 of 18 recipients after 8 Gy (days 31, 37 and 41) of TBI. Isolated MHC class II disparity led to the death of 3 of 20 animals after 10 Gy (days 32, 34 and 41) and 2 of 9 after 8 Gy (days 39 and 43) of TBI. The rats developing lethal GvHD after 10 and 8 Gy were chimeric (92 +/- 4 and 67 +/- 9%, respectively) shortly before time of death. To examine the degree of GvHD in each group, a clinical score for rodents was applied as stated above. Hereby, serological MHC class II disparity was identified as most critical for GvHD development in the present rat model. The degree of GvHD increased around day 20 in groups with isolated MHC class II and complete MHC mismatches, despite highly effective *in vitro* α/β TCR^+^ cell depletion of donor bone marrow cells (**Fig 1A**). After isolated MHC class I or non-MHC mismatched BMT, no increase of GvHD scores over time was observed. Of note, the detected peak at day 0 was caused by the procedure of TBI itself.

To test the MHC class II dependent GvHD induction, bone marrow cells without *in vitro* α/β TCR^+^ cell depletion and additional 5 x 10^7^ donor lymph node cells were injected after 10 Gy of TBI. Upon engraftment, all animals receiving BMT with isolated MHC class II disparity died due to acute GvHD until day 16 after BMT, whereas none of the recipients with isolated MHC class I disparity developed clinical manifest GvHD symptoms according to the selected criteria (**Fig 1B**).

### Matching MHC class II antigens of allogeneic heart grafts to donor bone marrow cells in stable chimeras ensures donor-specific tolerance

Before testing alloreactivity against solid organ grafts, high-grade stable chimeras (conditioned with 10 Gy of TBI) were analyzed by flow cytometry for their reconstitution patterns in peripheral blood on day 100 after BMT. Absolute numbers of leukocytes and their main subpopulations (T-, B- and NK-cells and granulocytes) did not significantly differ between chimeric recipients and untreated controls of naïve LEW rats (**Table 3**).

**Table 3:** Blood leukocyte reconstitution on day 100 after BMT (10 Gy).

MHC disparity	Leukocytes	T cells	B cells	NK cells	Granulocytes
		α/β TCR^+^ (R73)	CD45RA^+^ (OX33)	NKR-P1^high^ (3.2.3.)	(His48^high^)
Naïve recipient	8133 +/- 814	4137 +/- 645	1197 +/- 299	409 +/- 30	756 +/- 169
Complete	9620 +/- 851	4495 +/- 435	1637 +/- 269	484 +/- 63	1463 +/- 128
MHC II	8980 +/- 705	3965 +/- 612	1061 +/- 166	543 +/- 77	1766 +/- 350
MHC I	10340 +/- 826	4350 +/- 434	1497 +/- 41	535 +/- 20	1324 +/- 314
non-MHC	9246 +/- 528	4173 +/- 531	1387 +/- 105	517 +/- 46	1645 +/- 287

NK cells, Natural killer cells; MHC, Major histocompatibility complex. Naïve rats (LEW.1AR2) with same gender and similar age were analyzed as controls.

To evaluate donor-specific tolerance induction of reconstituted high-grade chimeras, heart grafts syngeneic to either the bone marrow recipient or donor strain as well as grafts from a 3^rd^ party strain (Brown Norway) were heterotopically transplanted on day 100 post BMT.

In addition, chimeras with isolated MHC class I or II donor-/recipient disparity were engrafted with hearts of donors, which were matched to the bone marrow donor strain either by MHC class I or MHC class II, respectively, to evaluate the role of MHC antigens for tolerance induction in stable chimerism (partially MHC matched heart grafts) (**Table 4**).

**Table 4:** MHC (RT1) immunogenetics of strain combinations for heart transplantation.

MHC match (Heart to BM recipient or donor)			MHC region		
	Strain combinations		RT1A	RTA1B/D	RT1C/E
			(class I)	(class II)	(class I)
**complete (recipient)**	Recipient	LEW.1A	**a**	**a**	**a**
	BM donor	LEW.1W	u	u	u
	Heart donor	LEW.1A	**a**	**a**	**a**
**complete (donor)**	Recipient	LEW.1A	a	a	a
	BM donor	LEW.1W	**u**	**u**	**u**
	Heart donor	LEW.1W	**u**	**u**	**u**
**class II (donor)**	Recipient	LEW.1AR2	a	a	u
	BM donor	LEW.1AR1	a	**u**	u
	Heart donor	LEW.1WR1	u	**u**	a
**class I (donor)**	Recipient	LEW.1WR1	u	u	a
	BM donor	LEW.1AR1	**a**	u	**u**
	Heart donor	LEW.1AR2	**a**	a	**u**
**3**^**rd**^ **party**	Heart donor	Brown Norway	n	n	n

BM, bone marrow; MHC, Major histocompatibility complex. Matched MHC loci of heart and BM recipients and donors, respectively, are shown in bold and underlined.

**Fig 2:**
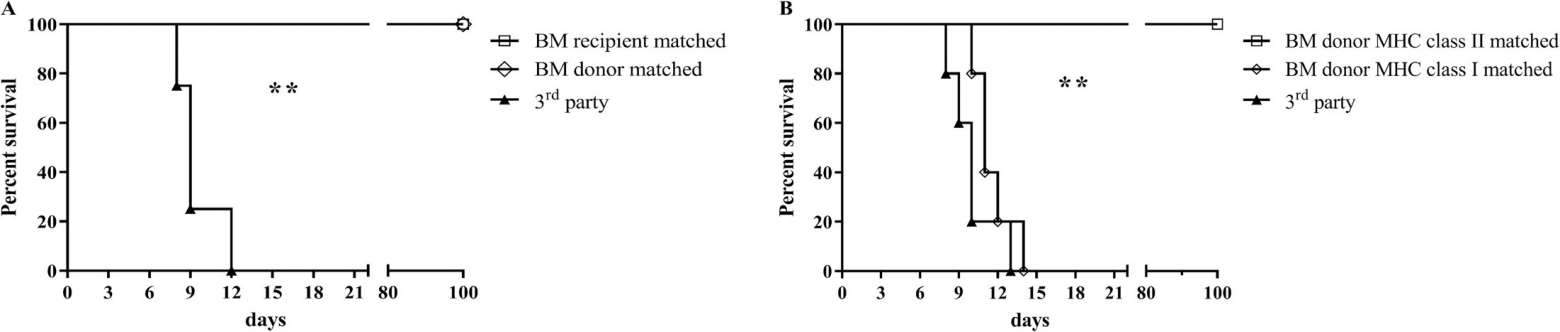
Survival of heart grafts transplanted to stable chimeras after BMT. (A) Heart grafts completely matched for MHC of the bone marrow recipients or donors were not rejected over the 100-day follow-up period, whereas rejection of 3^rd^ party grafts was still observable. (B) Partial matching of heart grafts to bone marrow grafts for MHC class II revealed donor-specific tolerance, whereas MHC class I matching did not prolong graft survival (for strain combinations depicted in Fig 2A/B see Table 4).

Chimeras showed tolerance towards hearts grafts syngeneic to the bone marrow recipient or donor, whereas immune competence against 3^rd^ party grafts was still observable (**Fig 2A and S1 Table**). Heart grafts, which were only partially MHC matched with the bone marrow donor strain, were accepted indefinitely only in case of matched MHC class II. Matching of MHC class I antigens between bone marrow and heart graft donors did not significantly prolong the course of rejection when compared to 3^rd^ party control grafts (**Fig 2B** and **S1 Table**). Rejected grafts were predominantly infiltrated by CD4^+^ T cells. Functioning grafts showed only marginal cell infiltration, especially with regard to CD25^+^ (activated) lymphocyte subsets (**S1A–S1D Fig**).

## Discussion

Establishing tolerance to allogeneic grafts, such as solid organs, is still the key issue in transplantation immunology. Apart from immunosuppressive regimens enabling acceptance to foreign tissue, the use of mixed hematopoietic chimerism after allogeneic BMT has proved to be sufficient in facilitating acceptance towards solid organ grafts in experimental settings. However, application in the clinical routine has not yet been established [18].

In this study we investigated influences of serological MHC disparities on BMT and establishment of stable mixed hematopoietic chimerism in well-defined congeneic rat strains. Successful induction of mixed chimerism after myeloablative TBI was observed across all chosen MHC (and non-MHC) disparities. As expected, the number of deaths due to GvHD was significantly elevated when compared to non-myeloablative conditioning–especially in donor-recipient combinations with full or isolated MHC-class II disparity. This observation was further accentuated after BMT without depletion of donor T cells and additional transplantation of donor lymphatic cells. Similar observations have been made in murine and porcine models of acute GvHD [19–21].

With respect to the significant morbidity and mortality after myeloablative TBI, we additionally analyzed the effects of myelosuppressive TBI and MHC disparity on outcome of recipients in terms of stable chimerism and incidence of GvHD.

In a murine model preconditioning via irradiation dosages of 1 Gy showed selective stem cell toxicity and significant depletion, enabling quantitative competition between recipient and donor-derived hematopoietic stem cells during the engraftment process, without development of myeloablation. The subsequent application of high bone marrow cell numbers (40 x 10^6^ per recipient) in a syngeneic setting (male to female) resulted in stable high-grade mixed chimerism [22].

In the present study a non-myeloablative TBI dosage of 6 Gy enabled safe hematopoietic stem cell engraftment over a non-MHC (CD45) allobarrier using 100 x 10^6^ bone marrow cells per recipient, which represents only around 5% of total bone marrow cell number. Isolated donor-recipient mismatches of MHC class I led to partial induction of mixed chimerism, whereas MHC class II or complete MHC disparity did not enable mixed chimerism. These observations were confirmed by increasing TBI to a dosage of 8 Gy: Whereas isolated MHC class I disparity led to hematopoietic stem cell engraftment in 80% of the recipients, MHC class II or complete MHC mismatch revealed stable chimerism in only 29 and 20% of the recipients 100 days post BMT, respectively. Therefore, MHC class I disparity had significantly less influence on the development of stable chimerism in the present model. These findings are contrary to observations previously made by other investigators: In a study by Oaks and Cramer, in which serological immunogenetics of BMT were analyzed using a rat model, donor MHC class I or class II disparity did not lead to a different outcome regarding hematopoietic stem cell engraftment; however, preconditioning was performed by myelotoxic doses of busulfan [20].

Likewise Vallera et al. found no significant influence of isolated MHC class I or class II disparity on the hematopoietic stem cell engraftment rate in 5 to 6 Gy irradiated murine recipients [23]. Conversely, in a porcine model of bone marrow transplantation MHC class I matching proved to be essential for successful engraftment after TBI (9 and 11 Gy, respectively). Nonetheless, MHC class II mismatched BMT showed poor outcome, most probably due to GvHD (no T-cell depletion of bone marrow cells) [19].

Lately, studies with murine models have shown evidence for MHC class II-mediated acute GvHD induction by recipient non-hematopoietic antigen-presenting cells and increased stimulation of donor T cell responses, giving a reasonable explanation for our reported observations [24,25].

Apart from diverse experimental settings, animal models and preconditioning regimens, an explanation for these contrary results could be a special role of rat NK cells for the engraftment and rejection process of allogeneic bone marrow cells. Farnsworth et al. discovered that upon irradiation of rats with 9 Gy, NK cells made up a significant proportion of the regenerating host cell populations leading to the assumption of a relative radioresistance of NK cells [26]. In line with these results investigators demonstrated alloreactive host NK cells as obstacle for engraftment of donor hematopoietic stem cells in a well-defined intra-MHC congeneic rat system [27–29].

Surprisingly, myelotoxic dosages of TBI showed successful engraftment of donor hematopoietic stem cells in our present study, despite MHC class I disparity, leading to the assumption that rejection via non-self-recognition mediated by host NK cells is of minor importance in our setting.

As was recently outlined by Zinöcker et al. alloreactive donor NK cells in a MHC class I disparate setting could also facilitate engraftment and donor reconstitution and therefore could have positive influence on establishing chimerism [30].

The direct expression of MHC class II on primitive hematopoietic stem cells and a subsequent interaction with the host immune system does not seem to be a plausible explanation for our observations, since studies in mice and humans did not detect relevant expression of MHC class II antigens on primitive hematopoietic stem cells [31]. CD34^+^ progenitor cells show reduced MHC class II expression in humans [32]. Unfortunately, analysis for MHC class II expression could not be performed in our study, since defined hematopoietic stem cell markers are not established for the applied rat strains. Attempts using CD90 as hematopoietic stem cell and progenitor marker revealed that the expression of MHC class II on the transplanted hematopoietic stem cells is weak.

However, the transplantation of donor-derived antigen-presenting cells such as B cells, monocytes or dendritic cells expressing MHC class II and the migration of donor-derived lineages upon engraftment, especially in lymphoid tissues such as the thymus as has been shown by our previous work, could significantly alter the induction of stable chimerism by promoting tolerance through presentation of allogeneic donor antigens, as suggested by others in the past [1,8,13].

Finally, to evaluate the role of MHC class I or MHC class II antigens for solid organ tolerance in stable mixed chimerism, we transplanted heart grafts from donors differently matched to the MHC regions of bone marrow donor strains in reconstituted, high-grade chimeras. MHC class II disparity was determined as main obstacle for tolerance towards solid organs in this model. This is in accordance to previous studies and confirms the MHC class II antigen as an essential tool of establishing donor-specific tolerance [10,13]. Of note, in MHC class II mismatched chimeras displaying lower grades of chimerism after 8 Gy of TBI, isolated MHC class II matching between heart and BM donor did not result in indefinite graft survival.

As additional corroboration of our results, LeGuern et al. and Jindra et al. each discovered that gene-based transfer of donor-MHC class II to recipients of allogeneic cardiac or skin grafts, respectively, resulted in indefinite graft survival. The tolerogenic effect of the MHC class II gene therapy is most likely explained through induction of regulatory T cells [33,34]. Previous studies have concluded that a lack of recipient antigen presentation through MHC class II disparate donor dendritic cells significantly impairs induction of regulatory T cells and therefore promotes initiation of chronic GvHD [35]. Although, the induction of regulatory T cells was not of primary interest in the current study, results from our previous work with a MHC class II disparate heart transplantation rat model indicated that Sirolimus exerted tolerogenic properties, most likely by increasing the fraction of CD4^+^CD25^+^ regulatory T cells after irradiation [8,36].

As for the clinical relevance of this work, several authors have found strong evidence for the importance of HLA class II matching in bone marrow and solid organ transplantation. Nonetheless, the underlying mechanisms are still a matter of debate and require basic immunological research in animal models [37–39].

## Conclusions

We were able to define MHC class II as major obstacle for successful development of chimerism after BMT and subsequent donor-specific solid organ tolerance. Furthermore, MHC class II disparity significantly increased the risk of GvHD in the present model. However, the underlying mechanisms of these observations remain unclear and need more investigations in the future, especially with a focus on the induction of allospecific regulatory T cells.

## Supporting information

S1 Fig Lymphocyte infiltration of heart grafts transplanted into high-grade chimeras. HPF, high-power field. BM, bone marrow. After 100 days (complete MHC and MHC class II match of heart and BM donor, respectively) or upon rejection (MHC class I match and no MHC match of heart and BM donor, respectively) heart grafts transplanted into high-grade chimeras were analyzed for lymphocyte infiltration.
(TIFF)

S1 Table Survival (in days) of heart grafts transplanted to stable high-grade chimeras 100 days after BMT. BM(T), bone marrow (transplantation); MHC, Major histocompatibility complex. Heart grafts were either completely matched for MHC of the bone marrow recipients or fully or partially matched for different haplotypes in the MHC of the bone marrow donors.
(PDF)
