# Supplementary figures and images for: The importance of MHC class II in allogeneic bone marrow transplantation and chimerism-based solid organ tolerance in a rat model
Source: PLoS One. 2020 May 22;15(5):e0233497. doi: 10.1371/journal.pone.0233497 (PMC7244129; doi:10.1371/journal.pone.0233497)

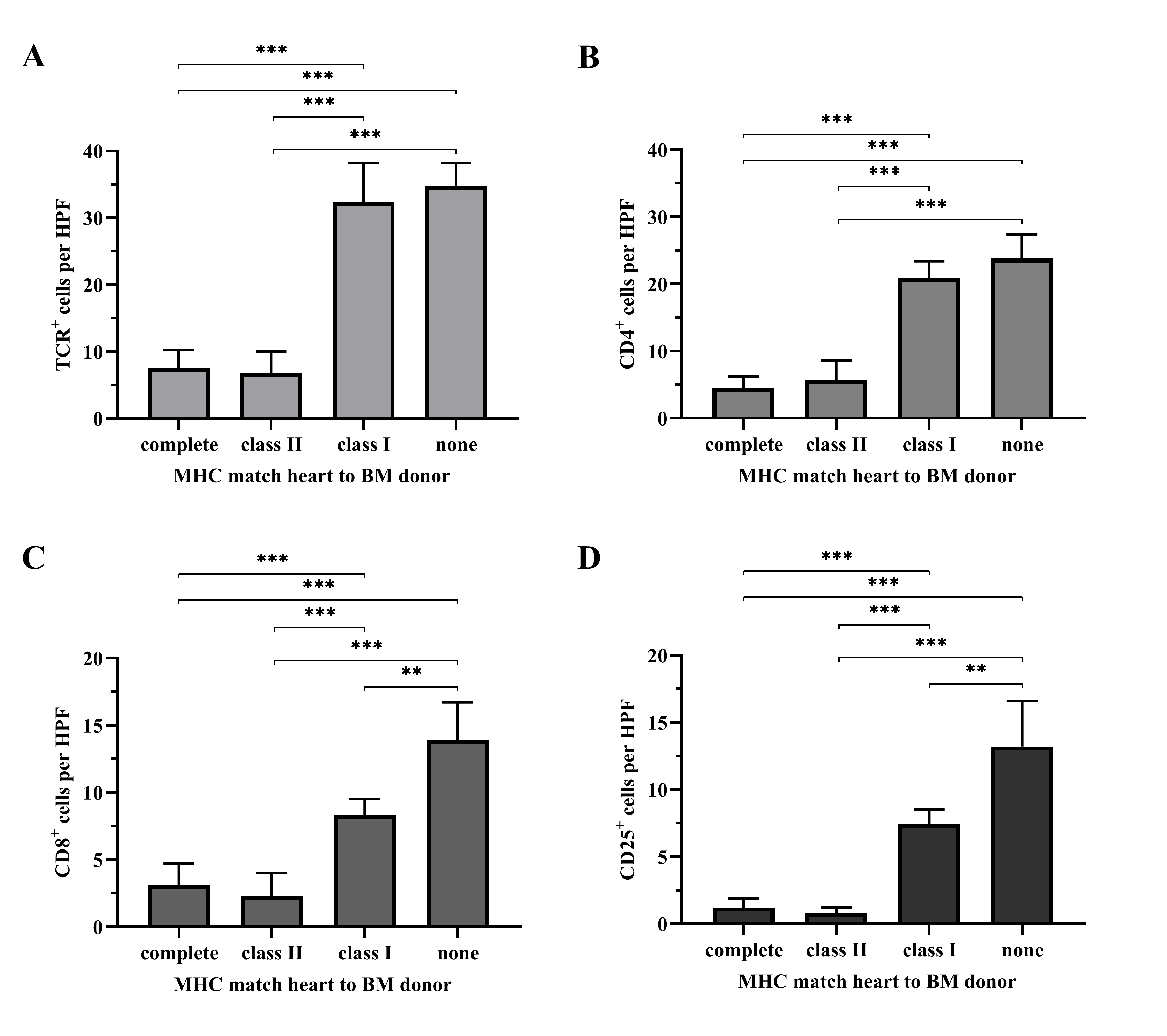

Supplement: S1 Fig — HPF, high-power field. BM, bone marrow. After 100 days (complete MHC and MHC class II match of heart and BM donor, respectively) or upon rejection (MHC class I match and no MHC match of heart and BM donor, respectively) heart grafts transplanted into high-grade chimeras were analyzed for lymphocyte infiltration. (TIFF) [file pone.0233497.s001.tiff]
