# Supplementary material for: The importance of MHC class II in allogeneic bone marrow transplantation and chimerism-based solid organ tolerance in a rat model
Source: PLoS One. 2020 May 22;15(5):e0233497. doi: 10.1371/journal.pone.0233497 (PMC7244129; doi:10.1371/journal.pone.0233497)
Supplement: S1 Table — BM(T), bone marrow (transplantation); MHC, Major histocompatibility complex. Heart grafts were either completely matched for MHC of the bone marrow recipients or fully or partially matched for different haplotypes in the MHC of the bone marrow donors. (PDF) [file pone.0233497.s002.pdf]

The importance of MHC class II in allogeneic bone marrow transplantation and chimerism-based solid organ tolerance in a rat model

Short title: MHC disparancy in bone marrow transplantation and induction of chimerism-based organ tolerance

Kai Timrott[1¶*], Oliver Beetz[1¶], Felix Oldhafer[1], Jürgen Klempnauer[1], Florian W. R. Vondran[1], Mark D. Jäger[1]

S1 Table. Survival (in days) of heart grafts transplanted to stable high-grade chimeras 100 days after BMT.

**MHC disparity for prior BMT**	**Heart transplantation in stable high-grade chimeras**				
	**BM recipient matched**	**BM donor matched**	**Partially BM donor matched**		**3rd party**
Complete	100, 100, 100, 100	100, 100, 100, 100	-		8, 9, 9, 12
MHC II	100, 100, 100	100, 100, 100, 100	100, 100, 100, 100, 100	(MHC II)	9, 10, 11
MHC I	100, 100, 100, 100	100, 100, 100, 100	10, 11, 11, 12, 14	(MHC I)	8, 9, 10, 10, 13
non-MHC	100, 100, 100, 100	-	-		9, 10, 12, 12, 13

BM(T), bone marrow (transplantation); MHC, Major histocompatibility complex. Heart grafts were either completely matched for MHC of the bone marrow recipients or fully or partially matched for different haplotypes in the MHC of the bone marrow donors
